# Staging of biliary atresia at diagnosis by molecular profiling of the liver

**DOI:** 10.1186/gm154

**Published:** 2010-05-13

**Authors:** Katie Moyer, Vivek Kaimal, Cristina Pacheco, Reena Mourya, Huan Xu, Pranavkumar Shivakumar, Ranajit Chakraborty, Marepalli Rao, John C Magee, Kevin Bove, Bruce J Aronow, Anil G Jegga, Jorge A Bezerra

**Affiliations:** 1 Division of Pediatric Gastroenterology, Hepatology and Nutrition of Cincinnati Children's Hospital Medical Center, 3333 Burnet Avenue, Cincinnati, OH 45229, USA; 2 Division of Pediatric Pathology, Cincinnati Children's Hospital Medical Center, 3333 Burnet Avenue, Cincinnati, OH 45229, USA; 3 Department of Environmental Health, University of Cincinnati College of Medicine, 231 Albert Sabin Way, Cincinnati, OH 45267, USA; 4 Department of Surgery of the University of Michigan Medical School, 1500 E. Medical Center Drive, Ann Arbor, MI 48109, USA; 5 Current address: Children's Pathology Services, Children's Hospitals and Clinics of Minnesota, 2525 Chicago Avenue, Minneapolis, MN 55404, USA; 6 Division of Biomedical Informatics, Cincinnati Children's Hospital Medical Center, 3333 Burnet Avenue, Cincinnati, OH 45229, USA

## Abstract

**Background:**

Young age at portoenterostomy has been linked to improved outcome in biliary atresia, but pre-existing biological factors may influence the rate of disease progression. In this study, we aimed to determine whether molecular profiling of the liver identifies stages of disease at diagnosis.

**Methods:**

We examined liver biopsies from 47 infants with biliary atresia enrolled in a prospective observational study. Biopsies were scored for inflammation and fibrosis, used for gene expression profiles, and tested for association with indicators of disease severity, response to surgery, and survival at 2 years.

**Results:**

Fourteen of 47 livers displayed predominant histological features of inflammation (N = 9) or fibrosis (N = 5), with the remainder showing similar levels of both simultaneously. By differential profiling of gene expression, the 14 livers had a unique molecular signature containing 150 gene probes. Applying prediction analysis models, the probes classified 29 of the remaining 33 livers into inflammation or fibrosis. Molecular classification into the two groups was validated by the findings of increased hepatic population of lymphocyte subsets or tissue accumulation of matrix substrates. The groups had no association with traditional markers of liver injury or function, response to surgery, or complications of cirrhosis. However, infants with an inflammation signature were younger, while those with a fibrosis signature had decreased transplant-free survival.

**Conclusions:**

Molecular profiling at diagnosis of biliary atresia uncovers a signature of inflammation or fibrosis in most livers. This signature may relate to staging of disease at diagnosis and has implications to clinical outcomes.

## Background

Biliary atresia results from a severe cholangiopathy that obstructs extrahepatic bile ducts, disrupts bile flow, and progresses to end-stage cirrhosis in most patients. Without knowledge of etiology and pathogenic mechanisms of disease, all patients are subjected to the same surgical and medical treatments despite the coexistence of different clinical forms. Thus, new strategies to phenotype the liver disease at diagnosis will aid the design of new clinical protocols that take into account the patient's biological makeup and facilitate studies of pathogenesis of disease. Among several proposed pathogenic mechanisms of disease [1,2], there is increasing evidence for an inflammatory response in promoting bile duct injury. For example, analysis of affected livers uncovered a prominent expression of proinflammatory genes and evidence of oligoclonal expansion of T lymphocytes at diagnosis [3-5]. The biological relevance of these findings was supported by mechanistic experiments demonstrating the roles of CD8+ lymphocytes or interferon-gamma in bile duct injury in a mouse model of biliary atresia [6-8]. In this mouse model, infection of newborn mice within the first 2 days of birth results in an inflammatory obstruction of extrahepatic bile ducts within 1 week and atresia by 12 to 14 days [9,10]. However, the extent to which individual cell types and molecular circuits relate to disease presentation and clinical course in humans is not well established.

Potential factors affecting the clinical course of children with biliary atresia include the center experience, age at portoenterostomy, and coexistence of embryonic malformations [11-17]. These factors notwithstanding, the progression of liver disease in most patients is the rule even after the surgical removal of atretic bile ducts and restoration of bile drainage, suggesting that biological factors operative at the time of portoenterostomy might influence the outcome of liver disease. Using histological approaches, previous studies linked the presence of inflammation [18] and fibrosis [19-21] with poor clinical outcome. Here, we aimed to determine whether molecular profiling of the liver identifies stages of disease at diagnosis. Analysis of liver biopsies uncovered a gene expression signature of inflammation or fibrosis that was associated with age at diagnosis and with differences in transplant-free survival.

## Methods

### Study population, covariates and outcomes

Tissue and clinical data were obtained from subjects enrolled into a prospective study of patients with biliary atresia evaluated at Cincinnati Children's Hospital Medical Center or into a multi-center prospective observational study carried out by the Biliary Atresia Research Consortium, with informed consent obtained from all infants' legal guardians. The study protocol conforms to the ethical guidelines of the 1975 Declaration of Helsinki and was approved by the human research committees of all participating institutions. Subjects were enrolled if diagnosed with biliary atresia and treated with portoenterostomy before 6 months of age. The diagnosis was defined by an abnormal intraoperative cholangiogram and histological demonstration of obstruction of extrahepatic bile ducts. Clinical and laboratory data were obtained at surgery and at 3- to 6-month intervals for the first 2 years of life (Additional file 1).

### Liver phenotyping

**Figure 1:**
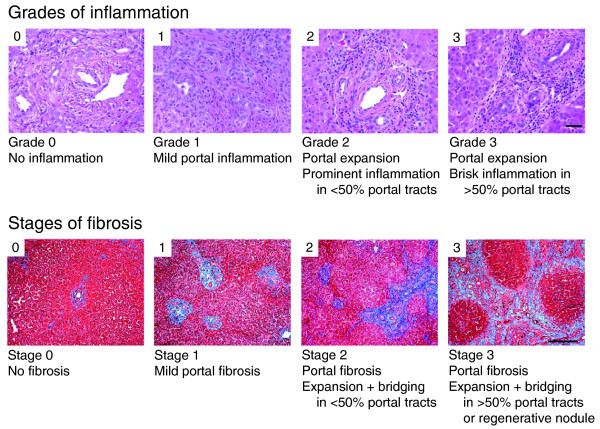
**Representative photographs of portal tracts stained with hematoxylin/eosin (upper panel) used for grading of liver sections in biliary atresia based on the presence of inflammatory cells (scale bar on photo 3 = 50 μm)**. The lower panels depict liver sections stained with trichrome for staging based on the extent of fibrosis (scale bar on photo 3 = 250 μm).

Wedge liver biopsies were obtained at the time of portoenterostomy and snap-frozen in liquid nitrogen, embedded frozen in OCT compound, or formalin fixed/paraffin embedded. Levels of inflammation were quantified by grading the population of portal tracts by inflammatory cells using liver sections stained with hematoxylin/eosin (graded 0 to 3 as described in Figure 1) and by counting cells immunostained with primary antibodies against CD3 (CD3 complex, Dako, Carpinteria, CA, USA; to identify T cells), CD11b and CD19 (EP1345Y and 2E2B6B10, respectively; both from Abcam, Cambridge, MA, USA; to identify B and myeloid cells, respectively), or CD56 (NCAM16.2, BD Biosciences, San Jose, CA, USA; to identify natural killer (NK) cells), with species-specific, fluorochrome-conjugated secondary antibodies according to published protocols [3,7,8]. To examine for fibrosis, sections were stained with trichrome and scored 0 to 3 according to a staging system published previously, with minor modifications (Figure 1) [19], and by consensus of two pathologists.

### Microarray and quantitative PCR

Genome-wide liver expression datasets were generated for individual subjects using pools of biotinylated cRNAs synthesized from 400 ng of total RNA isolated from 10 to 20 mg of frozen liver samples. cRNA pools were hybridized to oligonucleotide-based human HG-U133 Plus 2.0 Array (Affymetrix, Santa Clara, CA, USA) containing 54,681 probe sets, scanned, and monitored for specific signals with GeneChip^® ^Operating Software as described previously [3,22,23]. Affymetrix CEL files were imported into GeneSpring v7.3 (Agilent Technologies, Santa Clara, CA, USA) and subjected to Robust Multichip Average normalization. Detailed information on handling of liver biopsy samples, protocols for RNA labeling, chip hybridization and signals, internal controls, normalization procedures, and analysis of gene expression were deposited in Gene Expression Omnibus [GEO:GSE15235]. Quantitative PCR was done in a real-time Mx3000P thermocycler employing specific primers (Additional file 2) and established protocols [3,22,23].

### Molecular signatures

Using the GeneSpring platform, we performed standard 'per-gene' median normalization for the entire gene expression dataset. Using 14 samples that were grouped as either inflammation or fibrosis based on the differences in histological scores being ≥2, the levels of expression for individual probes were filtered based on fold change >2 between the two groups. This yielded 304 probesets, which were then subjected to a Welch's *t*-test, with a significance cutoff of 0.05 and Benjamini and Hochberg false discovery rate (FDR) multiple testing correction (5% FDR), generating a list of 150 probesets.

To evaluate the predictive ability of the 150-probeset signatures to identify inflammation or fibrosis, we applied the supervised method of prediction analysis of microarrays (PAM) [24-26]. In this approach, all genes are reassessed according to their ability to separate individual types; those genes that are less useful in discriminating between these types are eliminated. Classification accuracy was assessed by a method of ten-times ten-fold cross-validation using the R-Project and Bioconductor package MCRestimate [24-26]. Briefly, the training set is subdivided into ten equal parts. Nine parts are used for training, then employed to make class predictions on the tenth part, which is used as the test set. After each portion has been used as the test set once, the division into 10 parts is done again and the 10-fold cross-validation is repeated 10 times for a total of 100 runs (that is, class predictions are made on each sample exactly 10 times). We applied this approach to the remaining 33 unclassified samples and assigned them into groups of inflammation or fibrosis. The accuracy of this classification method was determined by finding a percent of samples correctly classified in at least six out of ten predictions made on the same sample as described previously [24-26].

### Functional analysis of genes

The genes highly expressed in the groups of inflammation or fibrosis were analyzed separately for functional themes using the Ingenuity Pathway Analysis 7.1 (Ingenuity Systems, Inc., Redwood City, CA, USA), using a right-tailed Fisher's exact test (with Benjamini-Hochberg/FDR correction) to evaluate for over-representation and displaying as -log(*P*-value); -log values exceeding 1.30 were significant (*P *< 0.05). Gene groups were also evaluated for biological relationship by searching for shared transcription factor binding sites (TFBSs) within 1 kb upstream of the transcription start sites of individual genes using Genomatix Gems Launcher [27], with a level of significance that includes FDR correction.

### Statistical testing of molecular signatures with clinical data

Testing for association between molecular signatures or histological groups with categorical variables (clinical form, cholangitis, ascites, transplant/death by 2 years of age) used Fisher's exact test. For quantitative dependent variables (age at diagnosis, level of bilirubin or alanine aminotransferase, weight Z score), means or medians were tested using Kruskal-Wallis one-way ANOVA on Ranks or two-sample Wilcoxon rank sum test (with continuity correction for age) when appropriate (two-sided *P*-values). The relationship of molecular signature or histological groups and age was assessed by the Gaussian Kernel method, while the relationship to outcome was examined by censored Kaplan-Meier. The R-package was used for all statistical analysis [28].

## Results

### Histological scoring

A total of 47 subjects were included in the study based on the availability of clinical data and tissue for analysis. Liver biopsies for individual subjects were examined for inflammation and the extent of fibrosis at diagnosis. For inflammation, we focused on the population of inflammatory cells within the portal space because it contains the primary site of biliary pathology and to avoid variables related to extra-medullary hematopoiesis that is commonly present in the hepatic lobule. We found that 34 of 47 (72.3%) of the biopsies had scores ≥1 for both inflammation and fibrosis (Additional file 3). Thus, we calculated the differences in scores within individual samples and identified biopsies displaying predominant features of either inflammation or fibrosis based on a differential score ≥2. Fourteen of 47 (30%) samples fell into this category, of which 9 had prominent inflammation and 5 had advanced fibrosis, while the remaining 70% had mixed histological features (differential scores <2). Next, we examined whether these 14 samples could be differentiated at the molecular level, and whether the signatures could identify other liver biopsies displaying molecular profiles for inflammation or fibrosis even when they were not evident by histology.

### Grouping by molecular signature

**Figure 2:**
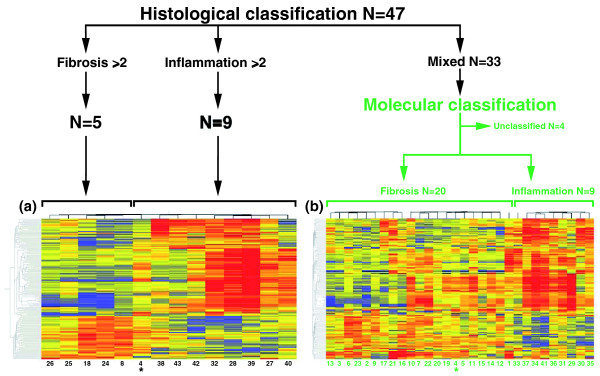
**Assignment of infants with biliary atresia into groups of inflammation or fibrosis at diagnosis**. When the differences in histological scores were ≥2, 5 of 47 livers had advanced fibrosis and 9 had predominant portal inflammation (black lines). These 14 livers displayed 150 gene probes that were differentially expressed between the fibrosis and inflammation groups (*P *< 0.05; Welch's *t*-test and 5% FDR - depicted as cluster analysis in **(a)**). Applying this expression signature to the 33 subjects classified histologically as 'mixed' (or unclassified), PAM assigned 29 subjects into groups of fibrosis or inflammation (N = 20 and N = 9, respectively) **(b)**. The cluster analyses depict gene expression as a color variation from red (high expression) to blue (low expression); yellow displays similar level between the groups. The numbers below the columns denote individual patients (listed in Additional file 3). *Patient 4 is included in both cluster analyses because PAM reclassified the liver into the fibrosis group.

Applying data filtering and two-way cluster analysis to the genome-wide expression data for the 14 liver biopsies, we identified 150 probe-sets with >2-fold differential expression (*P *< 0.05, 5% FDR; Figure 2a). This profile contained 115 unique genes, of which 77 were over-expressed in the inflammation group and 38 in the fibrosis group (Additional file 4). To examine whether the gene expression signature could be applied to individual samples and group them into inflammation or fibrosis profiles, we first applied the PAM-based 10-fold cross-validation method using the 150 probes against the entire gene expression dataset for each one of the 14 biopsies separately. Initial testing with PAM to identify a smaller set of gene probes that could best characterize each group showed that the removal of any gene probe increased misclassification errors in cross-validation. Therefore, using the entire set of 150 probes, PAM predicted the inflammation group in 8 of the 9 livers with predominant inflammation scores by histology; the remaining liver displayed a signature typical of the fibrosis group (infant 4 in Figure 2a, b). PAM also predicted the fibrosis group in all five biopsies previously classified as fibrosis based on predominant histological scores. We then used the same approach to group the 33 infants with mixed histological features (differential scores <2) into inflammation or fibrosis based on their gene expression profiles. From this cohort, 29 of 33 liver biopsies were classified as either inflammation or fibrosis. Interestingly, this classification was in agreement with 76% of the biopsies that had a differential histological score = 1 for inflammation or fibrosis (Additional file 3). Collectively, the addition of 33 biopsies to the 14 other biopsies (with a revised classification for biopsy 4 according to molecular profiling) grouped 43 of 47 (91%) infants into either molecular inflammation or fibrosis (Figure 2b). This pointed toward the potential existence of prominent biological processes at diagnosis that may be relevant to staging of disease.

### Testing of biological plausibility

**Figure 3:**
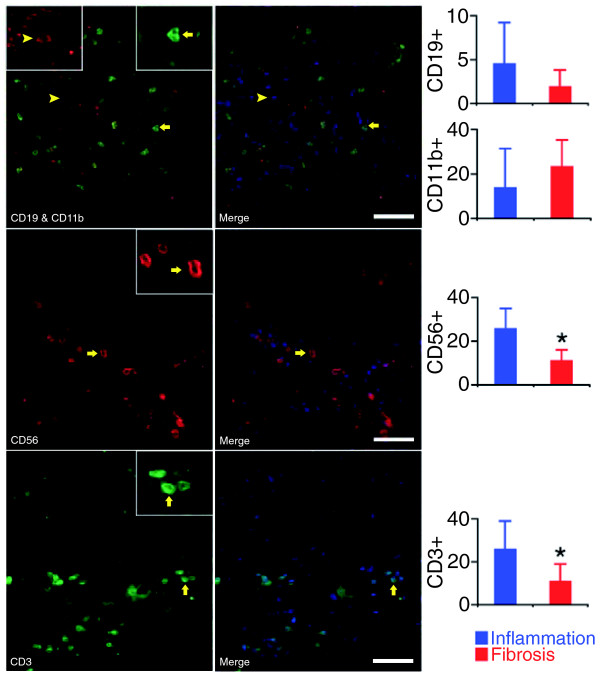
**Quantification of hepatic mononuclear cells in portal tracts**. Immunofluorescence panels (left) identify the population of portal tracts by B lymphocytes (CD19), myeloid cells (neutrophils and macrophages: CD11b), NK cells (CD56), and T cells (CD3) in livers with a molecular signature of inflammation. Photos on the right depict the left photos after nuclear staining with DAPI (white bar = 50 μm). The graphs on the right show the average number (± standard deviation) of stained cells in portal tracts from six livers with the inflammation signature and from five with the fibrosis signature. *P < 0.05.

To determine whether individual molecular signatures are supported by underlying biological processes and linked to pathogenesis of disease, we sought validation of the inflammation signature by quantifying the hepatic population by lymphocyte subtypes and myeloid cells (neutrophils and macrophages) using immunofluorescence. For these experiments, we only included liver biopsies that had a minimum of eight portal tracts per individual histological section in order to maximize the representation of the cell counts for each subject; the classification into the groups of inflammation or fibrosis was based on molecular profiles. Cell count showed an increase in T and NK lymphocytes in portal tracts of subjects in the inflammation group, which were approximately 2.4-fold more abundant than the fibrosis group (*P *< 0.05; Figure 3).

**Figure 4:**
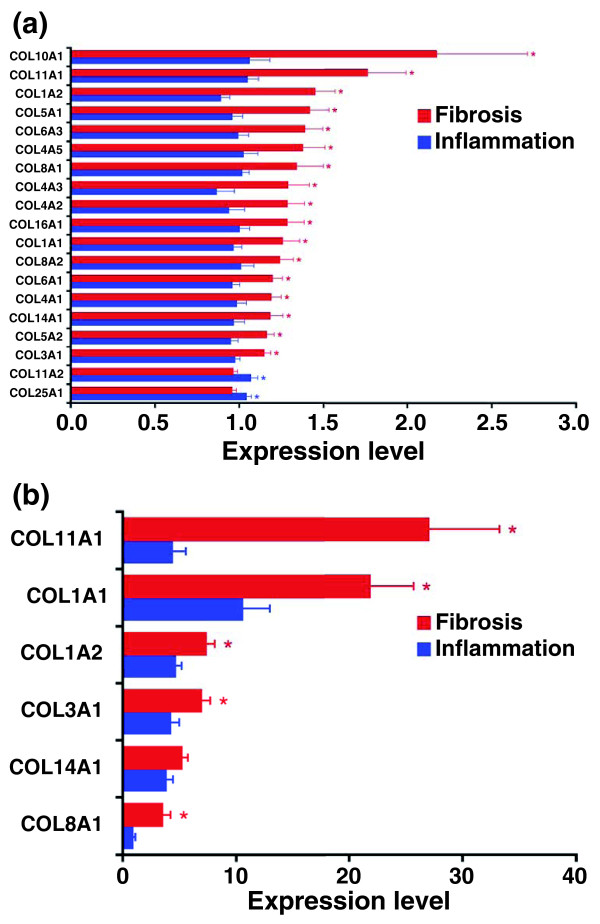
**Hepatic mRNA expression for collagen genes**. **(a) **Hepatic mRNA expression for collagen genes in subjects comprising the inflammation (N = 17) or fibrosis (N = 26) groups by microarrays. **(b) **mRNA expression by real-time PCR is shown for a subset of collagen genes shown in (a). Results are shown as mean ± standard error for individual genes as a ratio to *GAPDH*; *P < 0.05 (*P*-values range from 0.048 to 3.6 × 10^-7 ^in (a).

For the fibrosis group, the trichrome staining previously used to stage fibrosis in individual liver sections provided initial support for an accumulation of extracellular matrix in diseased livers. Seeking further validation, we determined the expression of several collagen genes that were not part of the 150-probe gene list and found a higher expression of several collagen-related genes in the fibrosis group (Figure 4a, b). These complementary approaches provided biological support for the use of gene expression profiling to classify biopsies into inflammation or fibrosis groups, and raised the possibility that the 150-probe set contains genes related to pathogenesis of disease.

**Figure 5:**
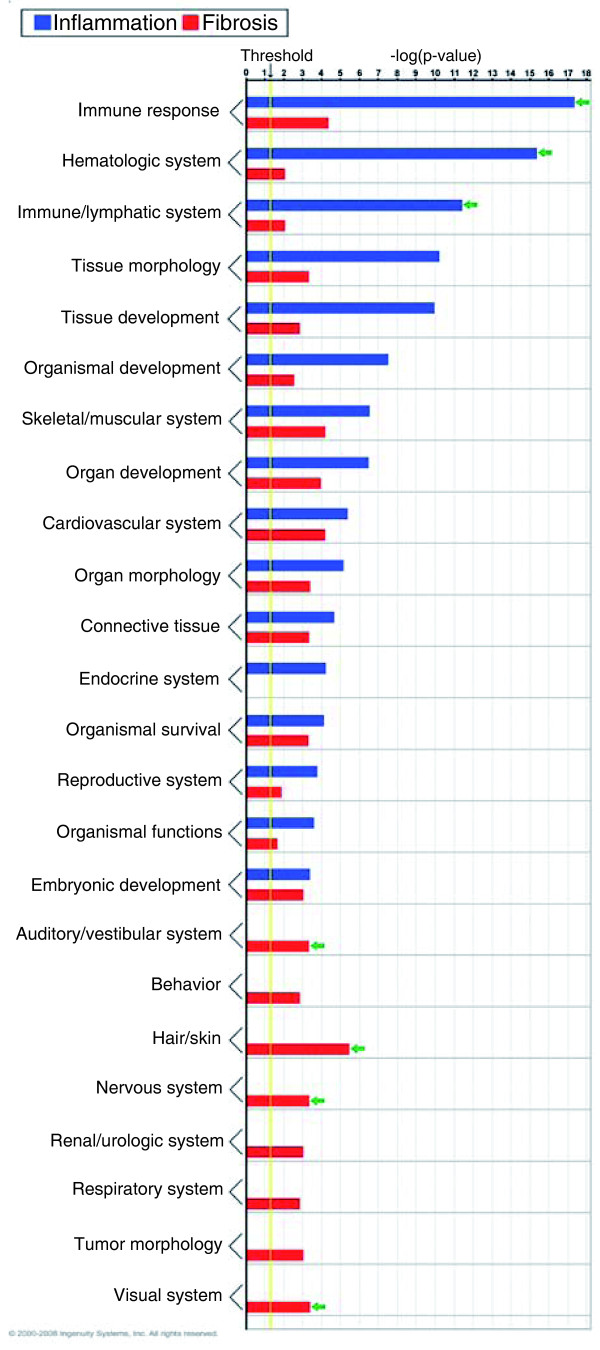
**Functional grouping of genes that are up-regulated in inflammation (N = 77 genes) or fibrosis (N = 38 genes) groups using Ingenuity Pathways Analysis**. Enrichment scores are represented as -log(*P*-value), with a threshold of 1.3 as the cut-off for significance (*P *< 0.05). Green arrows point to predominantly involved processes.

**Figure 6:**
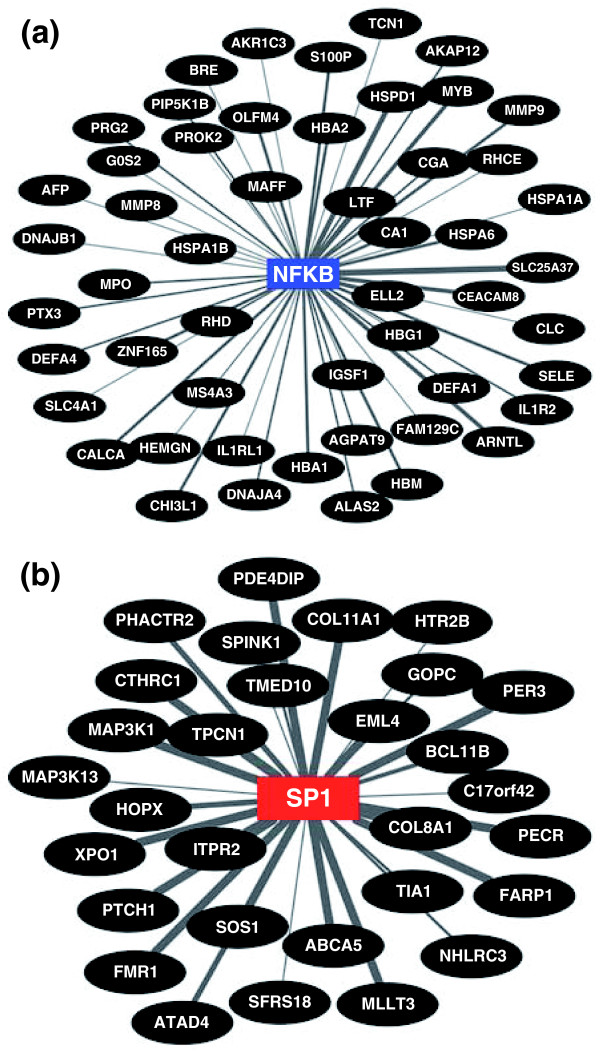
**Functional relatedness of genes overexpressed in subjects with (a) the inflammation signature with NFkB or (b) the fibrosis signature with SP1 based on the number of TFBSs**. The connecting line thickness is directly proportional to the number of TFBSs in the respective promoter regions. See Additional file 6 for the list of TFBSs for NFkB and SP1.

Functional analysis of the genes overexpressed in the inflammation group showed that three gene groups with the highest levels of statistical significance related to immune, hematological, and lymphatic systems, each with 24 to 26 genes (*P *< 0.001; Figure 5). Analyzing the TFBSs, genes that were up-regulated were functionally related to 49 transcription factors based on shared binding sites (Additional file 5). Among these transcription factors we highlight the sites for nuclear factor of activated T-cells (NFAT; 65 genes, *P *< 0.001) and nuclear factor (NF)kB (60 genes, *P *< 0.001; Figure 6a; Additional file 6) because both regulate immunity genes, but only the pleiotropic transcription factor NFkB being previously linked to biliary atresia [29,30]. Applying the same strategies for the 38 genes overexpressed in the fibrosis group, the functional groups with the highest levels of significance contained much fewer genes (2 to 7 per group, *P *< 0.049; Figure 5) which, surprisingly, were not related to matrix production/clearance. As a group, the genes identified only six transcription factors, which included E2F (38 genes, *P *< 0.001) and SP1 (38 genes, *P *= 0.001; Figure 6b; Additional file 6). E2F regulates cellular proliferation and TGFβ1-induced expression of matrix substrates, while SP1 is a potent inducer of extracellular matrix expression by fibroblasts [31-33], but neither has been linked to pathogenesis of biliary atresia.

### Testing for clinical relevance

To explore whether the molecular groups of inflammation or fibrosis have relevance to clinical presentation and/or progression of disease, we performed association tests between individual groups and clinical and laboratory parameters. We found no difference in sex, race, ethnic background, or clinical forms (perinatal or biliary atresia-splenic malformation) between the groups (Table 1). At the time of diagnosis, patients in both groups had similar degrees of hepatocellular injury or cholestasis (based on serum levels of alanine aminotransferases and bilirubin). There was no difference of serum bilirubin and nutritional status at 3 and 6 months after surgery, or in the percent of patients with episodes of cholangitis or ascites (Table 1).

**Table 1:** Relationship between clinical and biochemical characteristics and molecular groups of inflammation and fibrosis in infants with biliary atresia

Patient characteristic	Inflammation group, N = 15	Fibrosis group, N = 26	Total, N = 41^a^	*P*-value^b^
Sex, N (%)				
Female	6 (40)	12 (46)	18 (44)	0.8
Male	9 (60)	14 (54)	23 (56)	
Race, N (%)				
White	11 (73)	19 (73)	30 (73)	1.0
Black	1 (7)	1 (4)	2 (5)	1.0
Asian	2 (13)	1 (4)	3 (7)	0.5
Other	1 (7)	5 (19)	6 (15)	0.6
Ethnicity, N (%)				
Hispanic	1 (7)	1 (6)	5 (12)	0.6
Nonhispanic	14 (93)	25 (94)	36 (88)	
Age in days, median (25-75%)	55 (46.3-63)	71 (54-80)	65 (50-75)	<0.01
Clinical type				
BASM N (%)	2 (13)	3 (12)	5 (12)	1.0
Perinatal N (%)	13 (87)	23 (88)	36 (88)	
Mean CB at diagnosis^c^	4.9 ± 2.1	5.8 ± 2.4	5.5 ± 2.3	0.3
Mean ALT at diagnosis^c^	125 ± 83	154 ± 74	144 ± 78	0.3
Mean CB at 3 months after HPE^c^	1.7 ± 2.6	3.5 ± 5.3	2.8 ± 4.5	0.3
Weight Z-score at 6 months after HPE^c^	-1.3 ± 1.1	-1.7 ± 1.2	-1.5 ± 1.2	0.4
Presence of cholangitis, N (%)	8 (53)	18 (72)	26 (63)	0.5
Presence of ascites, N (%)	4 (27)	12 (48)	16 (40)	0.3

^a^Six patients from the cohort of 47 patients are not included because they were 'unclassified' by gene expression profiling (N = 4) or dropped off the study before 2 years of age (N = 2). ^b^*P*-values denote levels of statistical differences between the inflammation and fibrosis groups. ^c^Mean ± standard deviation. ALT, alanine aminotransferases; BASM, biliary atresia splenic malformation (polysplenia or asplenia); CB, conjugated bilirubin; HPE, hepatoportoenterostomy.

**Figure 7:**
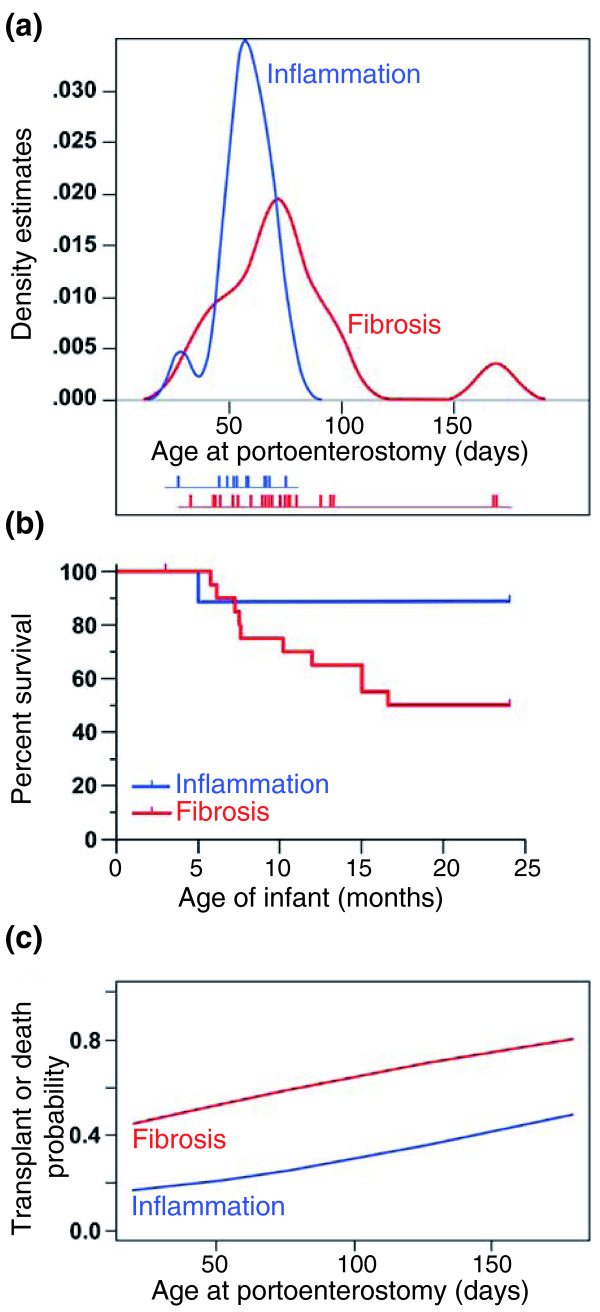
**Relationship between molecular groups and clinical features**. **(a) **The probability density function of age at the time of surgery (Kasai procedure) in relation to molecular signatures of inflammation or fibrosis in biliary atresia. The age of individual patients is shown below the graph as short vertical bars. **(b) **Kaplan-Meier analysis shows a decreased survival with the native liver in infants with the fibrosis signature (*P *= 0.04). **(c) **Logistic regression modeling depicts the effect of age on the association between molecular groups and the probability of transplant or death by 2 years of age (*P *= 0.079).

As a group, subjects in the inflammation group were younger than those in the fibrosis group at the time of diagnosis (median {25 to 75% ranges}: 55 {46.3 to 63} versus 71 {54 to 80}, *P *< 0.01). Although there was some overlap in age at diagnosis, a probability density function of age estimated by the Gaussian kernel method showed that the centers of distribution were not equal (*P *< 0.01), with several subjects with the fibrosis group diagnosed beyond 80 days (Figure 7a). The association between inflammation group and younger age at diagnosis raised the possibility that the presence of inflammation reflects an earlier stage of disease and may relate to clinical outcome. To examine this possibility, we tested the association between the two groups and clinical outcome at 2 years of age. We found that the fibrosis group was significantly associated with death or need for liver transplantation (odds ratio 8.2, 95% confidence interval 0.84 to 424, *P *= 0.04). Consistent with this finding, Kaplan-Meier survival analysis showed the fibrosis group was associated with a significantly lower transplant-free survival when compared to the inflammation group (*P *= 0.04; Figure 7b). Lastly, based on previous reports that age at portoenterostomy may influence long-term outcome, we performed binary logistic regression modeling and found that age alone at portoenterostomy did not influence outcome (*P *= 0.46), but the probability of death or need for transplant was influenced by molecular groups as a function of age at a *P*-value of 0.079 (Figure 7c).

**Figure 8:**
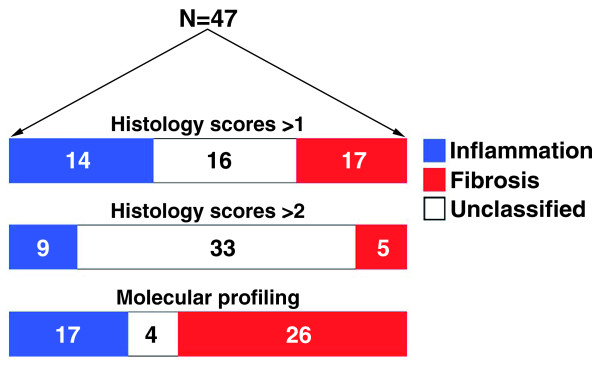
**Classification of 47 infants with biliary atresia into groups of inflammation or fibrosis based on differential histological scores ≥1 or ≥2 or on molecular profiling at diagnosis**.

It remained to be determined whether similar associations with age and transplant-free survival were present if the variables were compared to subjects that were grouped into inflammation or fibrosis using predominant histological features alone. To address this possibility, we grouped subjects according to the differences between inflammation and fibrosis scores for each subject (Additional file 3). From the entire cohort of 47 subjects, 14 (30%) had a differential score of ≥1 for inflammation and 17 (36%) for fibrosis; the remaining 16 (34%) were unclassified due to the differences between inflammation and fibrosis being zero (Figure 8). Comparative analysis between the inflammation and fibrosis groups showed no differences in patient demographics, clinical forms (perinatal or biliary atresia-splenic malformation), mean serum levels of conjugated bilirubin or alanine aminotransferases at diagnosis or at 3 months after hepatoportoenterostomy, weight Z-score, or incidence of cholangitis or ascites (Table 2). We also found no relationship of either group with age at diagnosis (*P *= 0.7; Additional file 7a) or difference in survival with the native liver at 2 years of age (*P *= 0.48, Additional file 7b). Thus, grouping of subjects based on differences between histological scores did not show relationships with clinical forms of disease, age at presentation, level of cholestasis at diagnosis or after portoenterostomy, or need for transplantation by 2 years of age.

**Table 2:** Relationship between clinical and biochemical characteristics and histological groups in infants with biliary atresia

Patient characteristic	Inflammatory subtype, N = 14	Fibrosing subtype, N = 17	Total, N = 31^a^	P-value^b^
Sex, N (%)				
Female	5 (36)	6 (35)	11 (35)	1.0
Male	9 (64)	11 (65)	20 (65)	
Race, N (%)				
White	11 (79)	16 (94)	27 (87)	1.0
Black	0 (0)	0 (0)	0 (0)	1.0
Asian	2 (14)	1 (6)	3 (10)	1.0
Other	1 (7)	0 (0)	1 (3)	1.0
Ethnicity, N (%)				
Hispanic	1 (7)	2 (12)	3 (10)	1.0
Nonhispanic	13 (93)	15 (88)	28 (90)	
Age in days, median (25-75%)	63 (55-65)	66 (51-77)	63 (51-73)	0.3
Clinical type				
BASM N (%)	1 (7)	1 (6)	2 (7)	1.0
Perinatal N (%)	13 (93)	16 (94)	29 (93)	
Mean CB at diagnosis^c^	5.1 ± 1.6	5.8 ± 2.5	5.6 ± 2.2	0.5
Mean ALT at diagnosis^c^	196 ± 150	192 ± 120	194 ± 136	1.0
Mean CB at 3 months after HPE^c^	2.3 ± 3.7	3.0 ± 3.5	2.6 ± 3.6	0.7
Weight Z-score at 6 months after HPE^c^	-1.1 ± 0.9	-1.8 ± 1.9	-1.4 ± 1.4	0.3
Presence of cholangitis, N (%)	5 (56)	10 (59)	15 (58)	1.0
Presence of ascites, N (%)	4 (36)	9 (53)	13 (47)	0.7

^a^Sixteen subjects from the cohort of 47 patients are not included because the differences in histological scores for inflammation and fibrosis were zero. ^b^*P*-values denote levels of statistical differences between inflammation and fibrosis groups. ^c^Mean ± standard deviation. ALT, alanine aminotransferases; BASM, biliary atresia splenic malformation (polysplenia or asplenia); CB, conjugated bilirubin; HPE, hepatoportoenterostomy.

## Discussion

We found that most livers of infants with biliary atresia display some elements of inflammation and fibrosis at diagnosis, with a subset (30% of the biopsies) containing more predominant histological features of either inflammation or fibrosis based on a greater differential score for the phenotypes. Using a gene expression signature highly specific for this subset of livers, we were able to group 91% of the biopsies into molecular inflammation or fibrosis and found significant association with age at portoenterostomy and transplant-free survival. These findings suggest that molecular profiling at diagnosis may stage the liver disease by the identification of biological pathways that may not be easily distinguishable by standard histological approaches to quantify inflammation or fibrosis. This may be due to intrinsic limitations of morphological methods (that is, hematoxylin/eosin or trichrome staining) or to a sampling artifact caused by a non-uniform tissue injury that varies between anatomical lobes and, perhaps more importantly, among neighboring lobules and portal tracts. Both obstacles may be overcome by the molecular profiling described herein. First, it uses RNA isolated from a fragment of tissue that, although small, contains a much larger representation of lobules/portal tracts than individual histological sections. Second, it is based on a molecular signature that contains the collective expression behavior of gene groups, without *a priori *bias related to their biological affiliations.

In experiments to validate the grouping of liver biopsies based on molecular signatures, we found that some gene groups are functionally related to the population of portal tracts by inflammatory cells and to molecular circuits previously implicated in pathogenesis of disease. For example, livers with a molecular signature of inflammation had an increase in the number of T and NK lymphocytes, overexpressed genes related to the immune system, and contained a cluster of genes with NFκB transcription sites. The activation of NFκB was also reported in this mouse model [29,30], but the enrichment of bindings sites for NFAT and other transcription factors in the list of genes that are differentially expressed suggests that molecular networks regulated by these factors may be important for the pathogenesis of disease. Despite the activation of these molecular pathways within the inflammation signature, we recognize that there might be distinctions between wedge and core liver biopsies. We were unable to make a direct comparison between these two types of biopsies due to the unavailability of tissues. Further, the isolation of RNA from a liver biopsy fragment may limit the potential implication of the findings with regards to disease pathogenesis because the biopsy includes several cell types and different regions of the liver lobule. This type of study will benefit from the use of laser-capture microdissection, which enables the analysis of specific cell types or anatomical regions (that is, portal tract versus lobule).

Gene expression profiling increases the number of available methods to quantify prominent biological processes in biliary atresia. A previous study used histological staining methods and reported that a high degree of syncytial giant cells, focal and bridging necrosis, and inflammation were associated with poor clinical outcome [18,19]. These findings differ from the improved outcome of our subjects assigned to the inflammation group, but we recognize that our findings will require validation in a larger population. Other studies have investigated the association of hepatic fibrosis and clinical course after portoenterostomy, with poor outcome reported for children with advanced fibrosis, either quantified by standard methods or aided by computerized morphometry [18-21]. This association was reproduced in our study in the children assigned to the group of molecular fibrosis.

The temporal differences in age at diagnosis for the molecular groups raise the possibility that the gene expression signatures reflect two distinct but inter-related stages of disease. The first stage, represented by younger patients with an inflammatory signature (most often but not exclusively at younger age), is placed biologically earlier in pathogenesis of disease, while the other patients may have transitioned to a more advanced stage of fibrosis. Such a continuum in the pathogenesis of disease has been demonstrated in the rotavirus-induced mouse model of biliary atresia [7,8,34], which begins with prominent inflammation of the liver and extrahepatic bile ducts and progresses to less inflammation and persistent duct obstruction; however, in humans, the stages appear not to obey a strict temporal organization. The presence of fibrosis in a subset of younger infants suggests that age alone cannot stage the disease. In these patients, the liver injury may have started at an earlier age, or it may have undergone rapid progression to fibrosis. The possibility of a rapid progression to fibrosis is supported by a previous report showing greater fibrogenesis in the livers of neonatal rats when compared to adults in a model of cholestasis induced by bile duct ligation [35].

Molecular profiling of liver biopsies at the time of diagnosis has been shown to differentiate the embryonic and perinatal forms of biliary atresia and identified genes with potential roles in pathogenesis of the embryonic form of disease [23]. For example, among the genes with unique expression patterns were five imprinted genes (*Igf2, Peg3, Peg10, Meg3*, and *IPW*) in infants with the embryonic form, suggesting that a failure to down-regulate embryonic gene programs may be involved in the non-hepatic malformations that are typical of this group of patients [23]. In a separate study, molecular profiling also revealed the activation of an interferon-gamma-rich proinflammatory circuit [3]. The biological relationship between this circuit and biliary injury was demonstrated in mechanistic studies showing that the *in vivo *depletion of interferon-gamma in mice prevented the obstruction of extrahepatic bile ducts [8]. Despite the informative data produced by these two studies, we recognize that the approach described here to stage the disease using molecular profiles needs future studies to evaluate its relevance in clinical practice and in potential therapies. This can be pursued by prospective validation in a new group of patients adequately powered for statistical analysis to look at clinical correlates and at responses to clinical intervention. For example, will the 150-probe set be reproduced if the same statistical method is applied to new livers with histological scores ≥2 for inflammation or fibrosis? Will infants with an inflammation signature do better if treated with anti-inflammatory drugs (for example, corticosteroids)? Formal answers to these questions will ultimately reveal the clinical impact of staging of liver disease and open opportunities for new trials that take into account the patient's biological makeup.

## Conclusions

Gene expression profiling of the liver at the time of diagnosis of biliary atresia identifies prominent signatures of inflammation or fibrosis in most patients. These signatures cannot be foreseen by traditional histological methods or by serum markers of liver injury or function. The segregation of inflammation with younger age at diagnosis and of fibrosis with decreased survival is in keeping with the ability of molecular profiling to stage the liver disease at diagnosis.

## Abbreviations

FDR: false discovery rate; NF: nuclear factor; NFAT: nuclear factor of activated T-cells; NK: natural killer; PAM: prediction analysis of microarray; TFBS: transcription factor binding site.

## Competing interests

The authors declare that they have no competing interests.

## Authors' contributions

KM built the patient database, performed immunostaining assays, analyzed the data and helped draft the manuscript; VK, HX, BJA, AGJ carried out the gene expression and informatics analyses and molecular staging of disease; CP and KB scored pathological analysis and fibrosis staining; RM and PS processed all liver tissue, participated in tissue sectioning, immunostaining, curing of the database, and data analysis; RC and MR performed statistical analyses; JM coordinated patient data acquisition, collection and processing of liver specimens, and tissue slides; JAB designed the study, analyzed the data, and drafted the manuscript.

## Supplementary Material

Additional file 1 **Data elements and rationale for inclusion in the study**. Click here for file

Additional file 2 **Description of PCR primers**. Click here for file

Additional file 3 **Description of subjects enrolled in the study**. Click here for file

Additional file 4 **Genes that are up-regulated in subjects with hepatic inflammation or fibrosis**. Click here for file

Additional file 5 **Transcription factors in subjects with hepatic inflammation or fibrosis**. Click here for file

Additional file 6 **Genes containing transcription factor binding sites for NFκB or SP1**. Click here for file

Additional file 7 **Figures of the relationship between histological groups with age and survival**. **(a) **The probability density function of age at the time of surgery (Kasai procedure) in relation to histological scores ≥1 for inflammation or fibrosis in biliary atresia (*P *= 0.7). The age of individual patient is shown below the graph as short vertical bars. **(b) **Kaplan-Meier analysis shows similar survival with the native liver in infants belonging to either group (*P *= 0.48). Click here for file
