# Supplementary material for: Staging of biliary atresia at diagnosis by molecular profiling of the liver
Source: Genome Med. 2010 May 13;2(5):33. doi: 10.1186/gm154 (PMC2887077; doi:10.1186/gm154)
Supplement: Additional file 1 — Data elements and rationale for inclusion in the study. [file gm154-S1.PDF]

Table S1

Data elements and rationale for inclusion in the study.

Type of data	Data element	Rationale
**Clinical data**	Age at diagnosis	Age of diagnosis may influence clinical outcome
	Clinical phenotype	
	-BASM[1] syndrome	Congenital malformation; severe course?
	-Perinatal biliary atresia[2]	Most common clinical form
	Conjugated bilirubin and ALT[3] at diagnosis	Indicators of impaired excretory function and liver injury
	Conjugated bilirubin 3 months after Kasai	High conjugated bilirubin is associated with poor outcome
	Weight 6 months after Kasai[4]	Malnutrition is associated with poor outcome
	Episodes of cholangitis	May promote progression of liver disease
	Presence of ascites	Clinical indicator of progressive liver disease
	Death or need for transplantation	Primary end-point indicating the severe phenotype
**Histology**	Hematoxilin/eosin staining	Analysis of inflammation of portal tracts
	Trichrome staining	Assessment of degree of fibrosis
**Immunostaining**	T cells	To quantify the population of portal tracts by mononuclear

	B cells	inflammatory cells
	NK cells	
	Macrophage/neutrophils	
**RNA**	RNA expression profile	To search for molecular basis of phenotypes

[1]BASM: biliary atresia-splenic malformation syndrome (polysplenia, asplenia)

[2]Biliary atresia without splenic malformation syndrome

[3]Alanine aminotransferase

[4]Kasai: Kasai procedure or hepatoportoenterostomy
