# Supplementary material for: Staging of biliary atresia at diagnosis by molecular profiling of the liver
Source: Genome Med. 2010 May 13;2(5):33. doi: 10.1186/gm154 (PMC2887077; doi:10.1186/gm154)
Supplement: Additional file 2 — Description of PCR primers. [file gm154-S2.PDF]

Table S2

Oligonucleotide primers, annealing temperatures, and PCR product sizes for amplified products.

Gene	Primer Sequences (Fibrosing Genes)	Annealing temp (°C)	Product size (bp)
COL11A1	For: 5'- GAAGAAAACCACGAAACCACTTG -3’<br>Rev: 5'- AAACTGCTGAATGTCCCCCTC -3’	50.9	121
COL1A1	For: 5'- GAGAGGAAGGAAAGCGAGGAG -3’<br>Rev: 5'- ACCAGCAACACCATCTGCG -3’	58.3	119
COL1A2	For: 5'- TGATGTTGAACTTGTTGCTGAGG -3’<br>Rev: 5'- GCGTGATGGCTTATTTGTTTTG -3’	51.1	127
COL3A1	For: 5'- TCCAGGATACCAAGGACCCC -3’<br>Rev: 5'- TGATTCTCCATCTTTTCCAGCAG -3’	55.7	112
COL14A1	For: 5'- CAGCAAAAGAAGTATGTAAGGCG -3’<br>Rev: 5'- TTCAGGGCTCCAACAGTGC -3’	51.9	127
COL8A1	For: 5'- TGGCAAAGAGTATCCACACCTACC -3’<br>Rev: 5'- GACCTTGTTCCCCTCGTAAACTG -3’	53.7	128
GAPDH	For: 5'- TGACATCAAGAAGGTGGTGAAGC -3’<br>Rev: 5'- TCAAAGGTGGAGGAGTGGGTG -3’	56.8	120

Temp: temperature
For: forward primer
Rev: reverse primer
