# Supplementary material for: Staging of biliary atresia at diagnosis by molecular profiling of the liver
Source: Genome Med. 2010 May 13;2(5):33. doi: 10.1186/gm154 (PMC2887077; doi:10.1186/gm154)
Supplement: Additional file 3 — Description of subjects enrolled in the study. [file gm154-S3.PDF]

Table S3

Clinical features at diagnosis, clinical course, and outcome in the first 2 years of age of infants with biliary atresia.

					At diagnosis			3 months after HPE	6 months after HPE			2 years of age
Sample	Clinical form	Infl [1]	Fib [1]	Molecular group	Age (days)	CB (mg/dL)	ALT (IU)	CB (mg/dL)	Weight Z score	Cholangitis (yes, no)	Ascites (yes, no)	Alive or transplant /death (months)
1	Perinatal	2	2	F	66	5.4	93	0.9	-2.28	No	No	Alive
2	Perinatal	2	2	F	97	3.9	114	0.8	-0.99	Yes	No	Alive
3	Perinatal	0	1	F	52	4.5	98	5.9	-2.91	Yes	No	Alive
4	Perinatal	3[2]	1[2]	F	91	4.5	208	0	-3.36	Yes	No	Alive
5	Perinatal	1	1	F	43	3.2	79	0	-1.39	Yes	No	Alive
6	Perinatal	1	2	F	80	5.9	230	0	0.80	Yes	No	Alive
7	Perinatal	1	2	F	77	3.3	159	0	-0.80	Yes	No	Alive
8	Perinatal	1[3]	3[3]	F	95	3.5	99	0.5	-2.70	Yes	No	Alive
9	Perinatal	1	2	F	65	3.5	95	0.2	N/A	Yes	No	Alive
10	Perinatal	2	3	F	46	8.7	262	0.4	0.09	No	No	Alive
11	Perinatal	1	2	F	73	6.8	164	N/A	N/A	Yes	Yes	Alive
12	Perinatal	2	2	F	75	5.9	233	N/A	N/A	N/A	N/A	N/A[4]
13	Perinatal	1	1	F	68	4.2	75	N/A	N/A	Yes	No	7.25
14	BASM	1	1	F	80	4.8	200	4.7	N/A	Yes	No	7.51
15	BASM	1	1	F	33	4.9	77	N/A	N/A	Yes	No	6.13
16	Perinatal	2	2	F	168	10.7	343	22	N/A	No	Yes	10.2
17	Perinatal	2	2	F	76	7.8	160	3.1	-3.00	No	Yes	11.97
18	Perinatal	0[3]	3[3]	F	68	9	253	N/A	N/A	Yes	Yes	6.85
19	Perinatal	0	1	F	54	4.7	124	4.8	-1.48	No	Yes	7.61

20	Perinatal	1	2	F	44	4.5	112	N/A	N/A	Yes	Yes	5.74
21	Perinatal	1	2	F	169	13	178	4.1	N/A	No	Yes	16.59
22	Perinatal	2	2	F	69	6.5	135	1.5	-1.50	Yes	Yes	15
23	Perinatal	1	1	F	97	7	134	0.5	-2.40	Yes	Yes	15
24	Perinatal	0[3]	2[3]	F	33	4.5	51	10.5	-0.63	Yes	Yes	7.28
25	Perinatal	1[3]	3[3]	F	73	6.1	249	N/A	-2.54	No	Yes	9.28
26	BASM	0[3]	3[3]	F	66	3.3	80	6.1	N/A	No	Yes	5.38
27	Perinatal	3[2]	0[2]	I[2]	45	3.1	54	3.3	0.15	Yes	No	Alive
28	Perinatal	3	1[2]	I[2]	73	N/A	94	N/A	-1.39	No	No	Alive
29	Perinatal	1	1	I	54	4	93	0.6	-0.50	Yes	No	Alive
30	Perinatal	1	1	I	55	3.9	29	0.4	-0.30	No	No	Alive
31	Perinatal	2	1	I	55	4	123	0	-2.24	No	No	Alive
32	Perinatal	2[2]	0[2]	I	63	7.1	324	0.1	-1.68	Yes	No	Alive
33	Perinatal	2	1	I	48	4.6	88	0.2	N/A	Yes	No	Alive
34	Perinatal	1	1	I	22	10.2	67	5.2	-0.01	Yes	No	Alive
35	Perinatal	2	1	I	63	5.4	171	0	-0.06	No	No	Alive
36	BASM	3	2	I	50	N/A	131	0.8	-1.63	Yes	No	Alive
37	BASM	0	0	I	41	3	19	0.1	-3.87	Yes	No	Alive
38	Perinatal	3[2]	1[2]	I	55	4.3	157	0	-2.18	N/A	Yes	N/A[4]
39	Perinatal	3[2]	0[2]	I	64	7.5	198	8.4	-1.41	No	Yes	8.85
40	Perinatal	3[2]	0[2]	I	65	3.1	247	3.2	-1.10	No	Yes	10.5
41	Perinatal	ND*	ND*	I	50	4.1	89	N/A	N/A	Yes	Yes	5.02
42	Perinatal	2[2]	0[2]	I	65	5.2	125	0.7	0.55	N/A	N/A	Alive
43	Perinatal	3[2]	1[2]	I	68	7.4	420	0	-1.03	N/A	N/A	Alive
44	Perinatal	1	0	U	75	4.7	409	11.2	-1.60	Yes	Yes	6.5
45	Perinatal	1	2	U	113	6.4	200	N/A	N/A	Yes	Yes	Alive
46	Perinatal	1	2	U	41	5.2	194	0.9	-5.59	No	No	Alive

47	BASM	1	2	U	51	4.8	717	N/A	N/A	No	No	5.51

HPE: Hepatoportoenterostomy; CB: conjugated bilirubin; BASM: biliary atresia splenic malformation; N/A: not available; I: inflammatory; F: fibrosing; U: unclassified; Histo: differential histological scores

[1]Numerical scores for inflammation (infl) or fibrosis (fib)

[2]Histological group of inflammation based on inflammation minus fibrosis scores ≥2

[3]Histological group of fibrosis based on fibrosis minus inflammation scores ≥2

[4]N/A: not available because the patient dropped off the study before 2 years of age

*ND: biopsy fragment too small to enable scoring for inflammation or fibrosis
