# Supplementary material for: Staging of biliary atresia at diagnosis by molecular profiling of the liver
Source: Genome Med. 2010 May 13;2(5):33. doi: 10.1186/gm154 (PMC2887077; doi:10.1186/gm154)
Supplement: Additional file 4 — Genes that are up-regulated in subjects with hepatic inflammation or fibrosis. [file gm154-S4.PDF]

Table S4

Genes that are up-regulated in the groups of inflammation (in blue) or fibrosis (in red) in children with biliary atresia, presented on descending order based on fold change between the subtypes.

Gene	Description	Fold Change	GO Biological Process Annotation
IL1RL1	Interleukin 1 receptor-like 1	5.617	Immune response<br>Signal transduction
DEFA1	Defensin, alpha 1	5.175	Defense response to bacteria<br>Defense response to fungi<br>Response to virus<br>Xenobiotic metabolism
S100A12	S100 calcium binding protein A12 (calgranulin C)	3.934	Defense response to bacteria<br>Defense response to fungi<br>Inflammatory response<br>Xenobiotic metabolism
HEMGN	Hemogen	3.561	---
HBG1	Hemoglobin, gamma A	3.521	Oxygen transport<br>Transport
RHAG	Rh-associated glycoprotein	3.482	Ammonium transport<br>Circulation<br>Protein complex assembly<br>Transport
HBM	Hemoglobin, mu	3.45	Oxygen transport<br>Transport
S100A8	S100 calcium binding protein A8 (calgranulin A)	3.415	Inflammatory response
CALCA	Calcitonin/calcitonin-related polypeptide, alpha	3.369	G-protein signaling, coupled to cAMP Nucleotide second messenger<br>Adenylate cyclase activation<br>Blood pressure regulation<br>Cell-cell signaling<br>Elevation of cytosolic calcium ion concentration<br>Phospholipase C activation<br>Skeletal development
ZNF165	Zinc finger protein 165	3.298	Regulation of transcription, DNA-dependent

DEFA4	Defensin, alpha 4, corticostatin	3.214	Defense response to bacteria<br>Defense response to fungi<br>Response to pest, pathogen or parasite<br>Xenobiotic metabolism
AKAP12	A kinase (PRKA) anchor protein (gravin) 12	3.132	G-protein coupled receptor signaling<br>Protein targeting<br>Signal transduction
ERAF	Erythroid associated factor	3.13	Hemoglobin metabolism<br>Hemopoiesis<br>Protein folding
GYPA	Glycophorin A (MNS blood group)	2.985	Biological process unknown
HBD	Hemoglobin, delta	2.933	Oxygen transport<br>Transport
CA1	Carbonic anhydrase I	2.922	One-carbon compound metabolism
S100P	S100 calcium binding protein P	2.91	---
SLC4A1	Solute carrier family 4, anion exchanger, member 1	2.874	Anion transport<br>Cell ion homeostasis
MS4A3	Membrane-spanning 4-domains, subfamily A, member 3 (hematopoietic cell-specific)	2.824	Signal transduction
CEACAM8	Carcinoembryonic antigen-related cell adhesion molecule 8	2.7	Immune response
HBA1	Hemoglobin, alpha 1	2.682	---
MAFF	V-maf musculoaponeurotic fibrosarcoma oncogene homolog F (avian)	2.622	Parturition<br>Regulation of transcription, DNA-dependent<br>Transcription from RNA pol II promoter
BRE	Brain and reproductive organ-expressed (TNFRSF1A modulator)	2.617	D-ribose metabolism<br>Carbohydrate metabolism
LTF	Lactotransferrin	2.61	Defense response to bacteria<br>Humoral immune response<br>Iron ion homeostasis<br>Iron ion transport<br>Transport

PROK2	Prokineticin 2	2.562	Activation of MAPK activity<br>Angiogenesis<br>Anti-apoptosis<br>Cell proliferation<br>Chemotaxis<br>Elevation of cytosolic calcium ion conc.<br>Inflammatory response<br>Neuropeptide signaling pathway<br>Smooth muscle contraction<br>Rhythmic process<br>Sensory perception of pain<br>Spermatogenesis
CRISP3	Cysteine-rich secretory protein 3	2.536	Cell-cell adhesion<br>Defense response<br>Fertilization<br>Innate immune response<br>Spermatogenesis
HSPA1B	Heat shock 70kDa protein 1B	2.449	mRNA catabolism<br>Protein folding<br>Response to unfolded protein
SPTA1	Spectrin, alpha, erythrocytic 1 (elliptocytosis 2)	2.395	Actin filament organization<br>Barbed-end actin filament capping<br>Regulation of cell shape
MPO	Myeloperoxidase	2.312	Anti-apoptosis<br>Defense response<br>Hydrogen peroxide catabolism<br>Response to oxidative stress
IL1R2	Interleukin 1 receptor, type II	2.29	Immune response
GYPB	Glycophorin B (MNS blood group)	2.286	---
CLC	Charcot-Leyden crystal protein	2.273	Antimicrobial humoral response (sensu) Vertebrata<br>Development<br>Lipid catabolism<br>Phospholipid metabolism
HBA2	Hemoglobin, alpha 2	2.229	---
RHCE	Rh blood group, CcEe antigens	2.224	Transport
ELA2	Elastase 2, neutrophil	2.201	Proteolysis
PTX3	Pentraxin-related gene, rapidly induced by IL-1 beta	2.188	Inflammatory response

ALAS2	Aminolevulinate, delta-, synthase 2 (sideroblastic/hypochromic anemia)	2.179	Biosynthesis<br>Heme biosynthesis
SLC25A37	Solute carrier family 25, member 37	2.179	Transport
HSPA6	Heat shock 70kDa protein 6 (HSP70B')	2.174	Protein folding<br>Response to unfolded protein
G0S2	G0/G1 switch 2	2.171	Regulation of progression through cell cycle
DNAJB1	DnaJ (Hsp40) homolog, subfamily B, member 1	2.167	Protein folding<br>Response to unfolded protein
XK	X-linked Kx blood group (McLeod syndrome)	2.146	Amino acid transport<br>Transport
C13orf18	Chromosome 13 open reading frame 18	2.145	---
MYB	V-myb myeloblastosis viral oncogene homolog (avian)	2.117	Regulation of transcription<br>Regulation of transcription, DNA-dependent
AFP	Alpha-fetoprotein	2.111	Immune response<br>Transport
RHD	Rh blood group, D antigen	2.096	---
AKR1C2	Aldo-keto reductase family 1, member C2 (dihydrodiol dehydrogenase 2; bile acid binding protein; 3-alpha hydroxysteroid dehydrogenase, type III)	2.09	Xenobiotic metabolism
CDH19	Cadherin 19, type 2	2.072	Homophilic cell adhesion
HSPA1A	Heat shock 70kDa protein 1A	2.037	Protein folding<br>Response to unfolded protein
BPI	Bactericidal/permeability-increasing protein	2.032	Defense response to bacteria<br>Immune response
DNAJA4	DnaJ (Hsp40) homolog, subfamily A, member 4	2.012	Protein folding
MMP9	Matrix metallopeptidase 9 (gelatinase B, 92kDa gelatinase, 92kDa type IV collagenase)	1.996	Collagen catabolism<br>Peptidoglycan metabolism<br>Proteolysis
HBB	Hemoglobin, beta	1.967	Biological process unknown<br>Oxygen transport<br>Transport
SELE	Selectin E (endothelial adhesion molecule 1)	1.905	Cell adhesion<br>Inflammatory response
SNCA	Synuclein, alpha (non A4 component of amyloid precursor)	1.901	Anti-apoptosis<br>Central nervous system development

CTSG	Cathepsin G	1.867	Immune response<br>Proteolysis
PRG2	Proteoglycan 2, bone marrow (NK cell activator, eosinophil granule major basic protein)	1.847	Defense response to bacteria<br>Inflammatory response
EPB42	Erythrocyte membrane protein band 4.2	1.832	Erythrocyte maturation<br>Peptide cross-linking<br>Regulation of cell shape
AGPAT9	Lysophosphatidic acid acyltransferase theta	1.817	Metabolism
ELL2	Elongation factor, RNA polymerase II, 2	1.815	RNA elongation from RNA pol II promoter<br>Regulation of transcription, DNA-dependent
DNTT	Deoxynucleotidyltransferase, terminal	1.805	DNA modification<br>DNA replication<br>Antimicrobial humoral response (sensu) Vertebrata
MMP8	Matrix metallopeptidase 8 (neutrophil collagenase)	1.789	Collagen catabolism<br>Peptidoglycan metabolism<br>Proteolysis
RNASE3	Ribonuclease, RNase A family, 3 (eosinophil cationic protein)	1.744	RNA catabolism<br>Defense response to bacteria
OLFM4	Olfactomedin 4	1.735	---
CHI3L1	Chitinase 3-like 1 (cartilage glycoprotein-39)	1.721	Carbohydrate metabolism<br>Chitin catabolism
TCN1	Transcobalamin I (vitamin B12 binding protein, R binder family)	1.714	Cobalamin transport<br>Cobalt ion transport<br>Ion transport
TRIM55	Tripartite motif-containing 55	1.634	Muscle development<br>Protein ubiquitination<br>Signal transduction
DNAJA1	DnaJ (Hsp40) homolog, subfamily A, member 1	1.593	Protein folding<br>Response to unfolded protein
HSPD1	Heat shock 60kDa protein 1 (chaperonin)	1.563	Cellular protein metabolism<br>Protein folding<br>Protein import into mitochondrial matrix<br>Response to unfolded protein
CAMP	Cathelicidin antimicrobial peptide	1.547	Defense response to bacteria<br>Response to pest, pathogen or parasite

CGA	Glycoprotein hormones, alpha polypeptide	1.511	Cell-cell signaling<br>Signal transduction
FAM129C	B-cell novel protein 1	1.508	---
PIP5K1B	Phosphatidylinositol-4-phosphate 5-kinase, type I, beta	1.475	---
IGSF1	Immunoglobulin superfamily, member 1	1.449	Cell adhesion
IGHM	Immunoglobulin heavy constant mu	1.43	Immune response
ARNTL	Aryl hydrocarbon receptor nuclear translocator-like	1.337	Circadian rhythm<br>Regulation of transcription, DNA-dependent<br>signal transduction
LOC654433	Hypothetical LOC654433	1.018	---
ACSM2B	Acyl-CoA synthetase medium-chain family member 2B	4.288	---
HTR2B	5-hydroxytryptamine (serotonin) receptor 2B	3.934	G-protein signaling, coupled to IP3 second messenger (phospholipase C activating<br>Circulation<br>Regulation of I-kB kinase/NF-kB cascade<br>Signal transduction
COL8A1	Collagen, type VIII, alpha 1	3.249	Cell adhesion<br>Phosphate transport
PTCH1	Patched homolog 1 (Drosophila)	2.682	Cell cycle<br>Cell proliferation<br>Morphogenesis<br>Negative regulation of cell cycle progression<br>Signal transduction
MAP3K13	Mitogen-activated protein kinase kinase kinase 13	2.565	JNK cascade<br>Activation of MAPKK activity<br>Activation of NFkB transcription factor<br>Protein amino acid autophosphorylation
CTHRC1	Collagen triple helix repeat containing 1	2.513	Phosphate transport
SPINK1	Serine peptidase inhibitor, Kazal type 1	2.497	---
PECR	Peroxisomal trans-2-enoyl-CoA reductase	2.366	---

COL11A1	Collagen, type XI, alpha 1	2.259	Cartilage condensation<br>Cell adhesion<br>Cell-cell adhesion<br>Extracellular matrix org. and biogenesis<br>Phosphate transport<br>Sensory perception of sound<br>Visual perception
GOPC	Golgi associated PDZ and coiled-coil motif containing	2.139	ER to Golgi transport<br>Golgi to plasma membrane transport<br>Protein transport
XPO1	Exportin 1 (CRM1 homolog, yeast)	2.139	mRNA export from nucleus<br>mRNA processing<br>Protein import into nucleus, docking<br>Protein transport
HOPX	HOP homeobox	2.121	Development<br>Regulation of transcription, DNA-dependent
FMR1	Fragile X mental retardation 1	2.109	---
TPCN1	Two pore segment channel 1	1.987	---
C8orf70	Chromosome 8 open reading frame 70	1.966	---
FMO2	Flavin containing monooxygenase 2 (non-functional)	1.948	Electron transport
C1orf41	Chromosome 1 open reading frame 41	1.913	---
NHLRC3	Similar to RIKEN cDNA 8030451K01	1.879	Insulin receptor signaling pathway
MLLT3	Myeloid/lymphoid or mixed-lineage leukemia (trithorax homolog, Drosophila); translocated to, 3	1.86	Regulation of transcription, DNA-dependent<br>transcription
ATAD4	ATPase family, AAA domain containing 4	1.853	Protein catabolism
ITPR2	Inositol 1,4,5-triphosphate receptor, type 2	1.842	---
TIA1	TIA1 cytotoxic granule-associated RNA binding protein	1.824	---
SOS1	Son of sevenless homolog 1 (Drosophila)	1.8	Ras protein signal transduction
C17orf42	Chromosome 17 open reading frame 42	1.689	---
CCDC76	Coiled-coil domain containing 76	1.687	---
BCL11B	B-cell CLL/lymphoma 11B (zinc finger protein)	1.636	Regulation of transcription, DNA-dependent

IFI44	Interferon-induced protein 44	1.616	Response to virus
PDE4DIP	Phosphodiesterase 4D interacting protein (myomegalin)	1.616	---
MAP3K1	Mitogen-activated protein kinase kinase kinase 1	1.613	---
LOC389831	Hypothetical gene supported by AL713796	1.597	---
PER3	Period homolog 3 (Drosophila)	1.597	Regulation of transcription, DNA-dependent<br>Signal transduction<br>Transcription
EML4	Echinoderm microtubule associated protein like 4	1.524	---
SFRS18	Chromosome 6 open reading frame 111	1.466	---
TMED10	Transmembrane emp24-like trafficking protein 10 (yeast)	1.465	---
PTP4A1	Protein tyrosine phosphatase type IVA, member 1	1.42	---
ABCA5	ATP-binding cassette, sub-family A (ABC1), member 5	1.406	---
PHACTR2	Phosphatase and actin regulator 2	1.284	Metabolism<br>Nucleobase, nucleoside, nucleotide and nucleic acid metabolism
FARP1	FERM, RhoGEF (ARHGEF) and pleckstrin domain protein 1 (chondrocyte-derived)	1.027	---
