# Supplementary material for: Staging of biliary atresia at diagnosis by molecular profiling of the liver
Source: Genome Med. 2010 May 13;2(5):33. doi: 10.1186/gm154 (PMC2887077; doi:10.1186/gm154)
Supplement: Additional file 5 — Transcription factors in subjects with hepatic inflammation or fibrosis. [file gm154-S5.PDF]

Table S5.

List of transcription factors identified based on the number of transcription factor binding sites (TFBSs) within 1 Kb region of the transcription start sites of individual genes. Also shown is the number of TFBSs in each gene (blue: up-regulated in the inflammation group; red: up-regulated in fibrosis group).

Transcription factor	Description	Number of genes	Number of binding sites	P value
PRDF	Positive regulatory domain I binding factor	66	121	2.20E-10
RREB	Ras-responsive element binding protein	54	94	1.07E-06
MAZF	Myc associated zinc fingers	63	185	9.35E-06
TALE	TALE homeodomain class recognizing TG motifs	58	77	1.05E-05
INSM	Insulinoma associated factors	50	73	1.99E-05
TBPF	TATA binding protein factor	79	341	6.03E-05
FAST	FAST-1 SMAD interacting proteins	56	95	6.59E-05
IKRS	Ikaros zinc finger family	58	100	1.31E-04
GFI1	Growth factor independence transcriptional repressor	49	78	1.51E-04
LHXF	Lim homeodomain factors	55	160	1.66E-04
NFAT	Nuclear factor of activated T-cells	65	134	1.66E-04
SNAP	snRNA-activating protein complex	60	120	1.83E-04
SMAD	Vertebrate SMAD family of transcription factors	50	83	2.42E-04
NKX6	NK6 homeobox transcription factors	56	136	3.07E-04
HOMF	Homeodomain transcription factors	70	246	3.38E-04
SF1F	Vertebrate steroidogenic factor	46	70	3.40E-04
NFKB	Nuclear factor kappa B/c-rel	60	125	4.47E-04

HEAT	Heat shock factors	73	191	5.06E-04
TEAF	TEA/ATTS DNA binding domain factors	46	63	6.99E-04
CDXF	Vertebrate caudal related homeodomain protein	47	90	7.17E-04
FKHD	Fork head domain factors	80	300	7.85E-04
EKLF	Erythroid krueppel like factor	63	192	0.00116
GATA	GATA binding factors	77	246	0.00129
GLIF	GLI zinc finger family	50	75	0.00143
MZF1	Myeloid zinc finger 1 factors	63	151	0.00158
RUSH	SWI/SNF related nucleophosphoproteins with a RING finger DNA binding motif	67	161	0.00218
NF1F	Nuclear factor 1	48	74	0.00248
EGRF	EGR/nerve growth factor induced protein C & related factors	65	247	0.00259
OCTP	Octamer binding protein	44	75	0.00282
RBPF	RBPJ - kappa	46	66	0.00392
HOXF	Paralog hox genes 1-8 from the four hox clusters A, B, C, D	84	508	0.00456
GKLF	Gut krueppel like factor	58	109	0.00566
EBOX	E-box binding factors	59	133	0.00671
SORY	SOX/SRY-sex/testis determinig and related HMG box factors	77	305	0.00857
BRNF	Brn POU domain factors	70	278	0.00908
CEBP	Ccaat/Enhancer Binding Protein	50	98	0.0109
CLOX	CLOX and CLOX homology (CDP) factors	77	244	0.0120
MYBL	Cellular and viral myb-like transcriptional regulators	70	131	0.0130
ETSF	Human and murine ETS1 factors	86	379	0.0139
SRFF	Serum response element binding factor	54	115	0.0172
ZFHX	Two-handed zinc finger homeodomain transcription	56	91	0.0188

	factors			
GREF	Glucocorticoid responsive and related elements	59	109	0.0233
NKXH	NKX homeodomain factors	80	320	0.0245
HNF1	Hepatic Nuclear Factor 1	50	83	0.0253
E2FF	E2F-myc activator/cell cycle regulator	64	155	0.0280
MEF2	MEF2, myocyte-specific enhancer binding factor	50	106	0.0314
STAF	Selenocysteine tRNA activating factor	45	62	0.0355
RORA	v-ERB and RAR-related orphan receptor alpha	56	88	0.0439
HOXC	HOX - PBX complexes	59	114	0.0492
MZF1	Myeloid zinc finger 1 factors	38	112	1.42E-05
EGRF	EGR/nerve growth factor induced protein C & related factors	38	219	8.17E-05
E2FF	E2F-myc activator/cell cycle regulator	38	132	4.44E-04
HEAT	Heat shock factors	39	85	7.86E-04
SP1F	GC-Box factors SP1/GC	38	188	0.00119
TBPF	TATA binding protein factor	39	125	0.00822
