# Supplementary material for: Staging of biliary atresia at diagnosis by molecular profiling of the liver
Source: Genome Med. 2010 May 13;2(5):33. doi: 10.1186/gm154 (PMC2887077; doi:10.1186/gm154)
Supplement: Additional file 6 — Genes containing transcription factor binding sites for NFκB or SP1. [file gm154-S6.PDF]

Table S6

List of genes containing transcription factor binding sites for NFκB or SP1, as depicted in Figure 7.

Transcription factor	Gene symbol	Gene name
**NFkB**	*AFP*	Alpha-fetoprotein
	AGPAT9	1-acylglycerol-3-phosphate O-acyltransferase 9
	AKAP12	A kinase (PRKA) anchor protein 12
	AKR1C3	Aldo-keto reductase family 1, member C3 (3-alpha hydroxysteroid dehydrogenase, type II)
	ALAS2	Aminolevulinate, delta-, synthase 2
	ARNTL	Aryl hydrocarbon receptor nuclear translocator-like
	BRE	Brain and reproductive organ-expressed (TNFRSF1A modulator)
	CA1	Carbonic anhydrase I
	CALCA	Calcitonin-related polypeptide alpha
	CEACAM8	Carcinoembryonic antigen-related cell adhesion molecule 8
	CGA	Glycoprotein hormones, alpha polypeptide
	CHI3L1	Chitinase 3-like 1 (cartilage glycoprotein-39)
	CLC	Charcot-Leyden crystal protein
	DEFA1	Defensin, alpha 1
	DEFA4	Cefensin, alpha 4, corticostatin
	DNAJA4	DnaJ (Hsp40) homolog, subfamily A, member 4
	DNAJB1	DnaJ (Hsp40) homolog, subfamily B, member 1
	ELL2	Elongation factor, RNA polymerase II, 2
	FAM129C	Family with sequence similarity 129, member C
	G0S2	G0/G1switch 2
	HBA1	Hemoglobin, alpha 1
	HBA2	Hemoglobin, alpha 2
	HBG1	Hemoglobin, gamma A

HBM	Hemoglobin, mu
HEMGN	Hemogen
HSPA1A	Heat shock 70kDa protein 1A
HSPA1B	Heat shock 70kDa protein 1B
HSPA6	Heat shock 70kDa protein 6 (HSP70B')
HSPD1	Heat shock 60kDa protein 1 (chaperonin)
IGSF1	Immunoglobulin superfamily, member 1
IL1R2	Interleukin 1 receptor, type II
IL1RL1	Interleukin 1 receptor-like 1
LTF	Lactotransferrin
MAFF	V-maf musculoaponeurotic fibrosarcoma oncogene homolog F (avian)
MMP8	Matrix metallopeptidase 8 (neutrophil collagenase)
MMP9	Matrix metallopeptidase 9 (gelatinase B, 92kDa gelatinase, 92kDa type IV collagenase)
MPO	Myeloperoxidase
MS4A3	Membrane-spanning 4-domains, subfamily A, member 3 (hematopoietic cell-specific)
MYB	V-myb myeloblastosis viral oncogene homolog (avian)
OLFM4	Olfactomedin 4
PIP5K1B	Phosphatidylinositol-4-phosphate 5-kinase, type I, beta
PRG2	Plasticity-related gene 2
PROK2	Prokineticin 2
PTX3	Pentraxin-related gene, rapidly induced by IL-1 beta
RHCE	Rh blood group, CcEe antigens
RHD	Rh blood group, D antigen
S100P	S100 calcium binding protein P
SELE	Selectin E
SLC25A37	Solute carrier family 25, member 37
SLC4A1	Solute carrier family 4, anion exchanger, member 1 (erythrocyte membrane protein band 3, Diego blood group)
TCN1	Transcobalamin I (vitamin B12 binding protein, R binder family)
ZNF165	Zinc finger protein 165

**SP1**	*COL8A1*	Collagen, type VIII, alpha 1
	TPCN1	Two pore segment channel 1
	SFRS18	Splicing factor, arginine/serine-rich 18
	MAP3K1	Mitogen-activated protein kinase kinase kinase 1
	EML4	Echinoderm microtubule associated protein like 4
	CTHRC1	Collagen triple helix repeat containing 1
	BCL11B	B-cell CLL/lymphoma 11B (zinc finger protein)
	GOPC	Golgi associated PDZ and coiled-coil motif containing
	MAP3K13	Mitogen-activated protein kinase kinase kinase 13
	HTR2B	5-hydroxytryptamine (serotonin) receptor 2B
	FMR1	Fragile X mental retardation 1
	FARP1	FERM, RhoGEF (ARHGEF) and pleckstrin domain protein 1 (chondrocyte-derived)
	XPO1	Exportin 1 (CRM1 homolog, yeast)
	MLLT3	Myeloid/lymphoid or mixed-lineage leukemia (trithorax homolog, Drosophila); translocated to, 3
	COL11A1	Collagen, type XI, alpha 1
	SOS1	Son of sevenless homolog 1 (Drosophila)
	HOPX	HOP homeobox
	TIA1	TIA1 cytotoxic granule-associated RNA binding protein
	PER3	Period homolog 3 (Drosophila)
	PDE4DIP	Phosphodiesterase 4D interacting protein
	TMED10	Transmembrane emp24-like trafficking protein 10 (yeast)
	SPINK1	Serine peptidase inhibitor, Kazal type 1
	C17orf42	Chromosome 17 open reading frame 42
	ABCA5	ATP-binding cassette, sub-family A (ABC1), member 5
	PHACTR2	Phosphatase and actin regulator 2
	ITPR2	Inositol 1,4,5-triphosphate receptor, type 2
	PECR	Peroxisomal trans-2-enoyl-CoA reductase
	PTCH1	Patched homolog 1 (Drosophila)
	NHLRC3	NHL repeat containing 3
	ATAD4	ATPase family, AAA domain containing 4
